# Correlation of Prolonged Corrected QT Interval With Ventricular Arrhythmias and In-Hospital Mortality Among ST-Elevation Myocardial Infarction Patients: A Mystique or Lucidity?

**DOI:** 10.7759/cureus.12356

**Published:** 2020-12-29

**Authors:** Muhammad Adnan Wattoo, Muhammad Tabassum, Kiran R Bhutta, Mehwish Kaneez, Syed Muhammad Jawad Zaidi, Hania Ijaz, Javeria Awan, Umer Irshad, Muhammad Junaid Azhar, Zainab Rafi

**Affiliations:** 1 Cardiology, Sialkot Medical College, Sialkot, PAK; 2 Internal Medicine, Islam Medical College, Sialkot, PAK; 3 Internal Medicine, Rawalpindi Medical University, Rawalpindi, PAK

**Keywords:** ventricular arrhythmias, in-hospital mortality, st-elevation myocardial infarction (stemi), prolonged qtc interval

## Abstract

Background

Ventricular arrhythmias (VAs) are a frequent cause of cardiovascular mortality, especially in developing countries. Prolongation of corrected QT (QTc) interval predisposes patients to life-threatening VAs. Our study aims to assess the correlation of prolonged QTc interval with VAs and in-hospital mortality among ST-elevation myocardial infarction (STEMI) patients.

Methods

This cross-sectional study analyzed the data from 40 patients with a confirmed diagnosis of STEMI and prolonged QTc interval. The patients were evaluated for several characteristics including their electrocardiography (ECG) findings. The frequency of in-hospital mortality and VAs developed after admission were recorded. Spearman correlation was used to assess the correlation of prolonged QTc interval with VAs and in-hospital mortality.

Results

Out of 40 cases, 30 patients were males and 10 were females with a mean age hovering at 52.95 ± 10.65 years. The mean QTc interval of our patients was 512.02 ± 49.74 milliseconds (ms). A total of 11 (27.5%) patients developed VAs while 14 (35%) of the patients succumbed to the disease complications. Spearman correlation showed a strong significant positive correlation of QTc interval with VAs (rho = 0.658, p < 0.001) and in-hospital mortality (rho = 0.314, p = 0.04).

Conclusion

Prolonged QTc interval is positively correlated with VAs and in-hospital mortality among STEMI patients. These patients should be regularly monitored and must be managed with caution as they have increased chances to develop VAs and in-hospital mortality. There is an utmost need for curation of guidelines that aid in risk stratification and appropriate management of such patients.

## Introduction

Ventricular arrhythmias (VAs) encompass a vast array of cardiac arrhythmias ranging from benign ventricular ectopic beats to fatal ventricular fibrillation [1]. VAs are attributed as one of the predominant causes of cardiovascular-related morbidity and mortality [2]. Ischemia leading to acute myocardial infarction causes momentous metabolic and electrophysiological alterations that ultimately predispose patients to frequent lethal VAs and irreversible myocardial injury [3]. Two of the most important morphological variants of acute myocardial infarction, as determined on electrocardiography (ECG), include non-ST-elevation myocardial infarction (NSTEMI) and ST-elevation myocardial infarction (STEMI) [3,4]. Additionally, a significant proportion of patients develop VAs in the setting of myocardial infarction [5]. These life-threatening VAs ultimately culminate into hemodynamic instability and the fatal demise of the patient. The morbidity and mortality due to VAs is declining in developed countries; however, its debilitating lethal manifestations are still a cause of major concern for cardiovascular morbidity and mortality in developing countries [6].

The predictive factors of acquiring VAs in STEMI include advancing age, comorbidities, biochemical changes, structural heart disease, and congenital or acquired prolonged QT interval [7]. QT interval is the time slice of ventricular excitation and recovery represented from the initiation of the QRS complex to the termination of T wave on the ECG. Furthermore, the variations in heart rate cause significant alterations in the length of the QT interval and as a result, it is adjusted using the Bazett formula (QT/√RR interval) and referred to as corrected QT (QTc) interval [6-8]. The standard QTc interval of below 450 milliseconds (ms) in males and 460 ms in females represents normal ventricular conduction [8]. On the contrary, the prolonged QTc represents the extended period of liability to progress to life-threatening arrhythmias. Furthermore, congenital or acquired prolonged QTc interval has also been associated with poor prognostic outcomes (thromboembolism, VAs, heart failure, and mortality) in STEMI patients [9].

Prolonged QTc interval is a potential cause of VAs and mortality in unstable angina and NSTEMI; however, the literature on QTc interval prolongation and STEMI related VAs is quite scarce [10,11]. Furthermore, prolonged QTc interval is also regarded as an independent and potent risk factor for lethal arrhythmias and mortality regardless of ischemic changes [11,12]. It has also been reported that QTc interval shortens initially in patients with STEMI [13]. The paucity of data and conflicting literature regarding the outcomes of prolonged QTc interval in STEMI makes our study an area of active medical research in the field of cardiology. Moreover, in developing nations like Pakistan, there is an unmet need to analyze the parameters that contribute to mortality in patients with prolonged QTc interval and STEMI. The current study, therefore, aims to assess the correlation of prolonged QTc interval with VAs and in-hospital mortality among STEMI patients. This will help in strengthening modern concepts and also open up new horizons for a series of researches and quality improvement projects.

## Materials and methods

This cross-sectional study was conducted in the Department of Cardiology, Imran Idrees Teaching Hospital, Sialkot, Pakistan from December 2019 till August 2020. Initially, a total of 277 STEMI patients were evaluated for prolonged QTc interval. STEMI patients with normal QTc interval, Wolf Parkinson’s White (WPW) syndrome, structural heart anomalies, hypothyroidism, chronic liver disease (CLD), chronic obstructive pulmonary disease (COPD), advanced malignancies, and end-stage renal disease (ESRD) were excluded from the study. Patients with electrolyte abnormalities (hypomagnesemia, hypocalcemia, and hyperkalemia) were also excluded as these electrolytes may alter the QTc interval. Additionally, patients on medications that prolong QTc interval (fluoroquinolones, macrolides, antihistamines, and atypical antipsychotics) were also excluded. Lastly, patients with a prior history of percutaneous coronary intervention (PCI), permanent pacemaker (PPM) implants, valvular replacement, and coronary artery bypass graft (CABG) surgery were also subjected to exclusion. After applying the above-mentioned rigorous exclusion criteria, a total of 40 patients qualified for the final analysis. The exclusion criteria ensured that only patients with STEMI and prolonged QTc interval are a part of the final study cohort. A senior cardiologist confirmed the ECG findings (QTc interval prolongation and ST-segment elevation) of each patient to eliminate the observer bias. High blood pressure and tachycardia of the patients were managed using antihypertensive agents (mainly sodium nitroprusside) and beta-blockers (mainly labetalol). Pertinently, thrombolytic therapy was employed to improve myocardial perfusion. Furthermore, patients were started on low molecular weight heparin for the initiation of antiplatelet action. Patients with poor oxygen saturation were given supplemental oxygen. 

The frequency of in-hospital mortality and VAs developed after admission was calculated. The data were collected from the computer records and patients’ files. During the data collection, consent was obtained from all participants, and confidentiality was ensured. The patients were studied for a myriad of demographic characteristics and ECG findings. Thereafter, the Spearman correlation was used to observe the correlation of QTc interval with VAs and in-hospital mortality. The data were eventually analyzed on IBM Statistical Package for Social Sciences (SPSS), version 25.0 (Armonk, NY). A p-value of less than 0.05 was considered statistically significant.

## Results

In the current study analyzing 40 cases, the mean age of the patients was 52.95 ± 10.65 years, with a range of 35-78 years. Table 1 elucidates the baseline demographic details of the study participants across gender, marital status, and smoking status.

**Table 1:** A breakdown of the baseline characteristics of the study participants.

Parameters		Frequency (n)	Percentages (%)
Gender	Males	30	75%
	Females	10	10%
Marital status	Unmarried	7	17.5%
	Married	33	82.5%
Smoking status	Smoker	26	65%
	Non-smoker	14	35%

The prevalence of a myriad of comorbidities among our study cohort is delineated in Table 2.

**Table 2:** The spectrum of various comorbidities among the patients. Some patients had more than one comorbid condition.

Comorbidities	Frequency (n)	Percentage (%)
Diabetes mellitus	17	42.5%
Hypertension	11	27.5%
Ischemic heart disease	29	72.5%
Chronic kidney disease (Stage 1-4)	4	10%
No comorbid condition	2	5%

The descriptive analysis of the ECG components of our patients is shown in Table 3.

**Table 3:** An elucidation of descriptive analysis of various ECG components among our patients. ECG: electrocardiography; ms: milliseconds; bpm: beats per minute; QTc: corrected QT

Main ECG finding	Mean value	Standard deviation	Range
QT interval (ms)	397.63	44.53	310-500
RR interval (ms)	641.21	134.98	450-1110
QTc interval (ms)	512.02	49.74	450-635
Heart rate (bpm)	98.09	18.98	52-135

**Figure 1:**
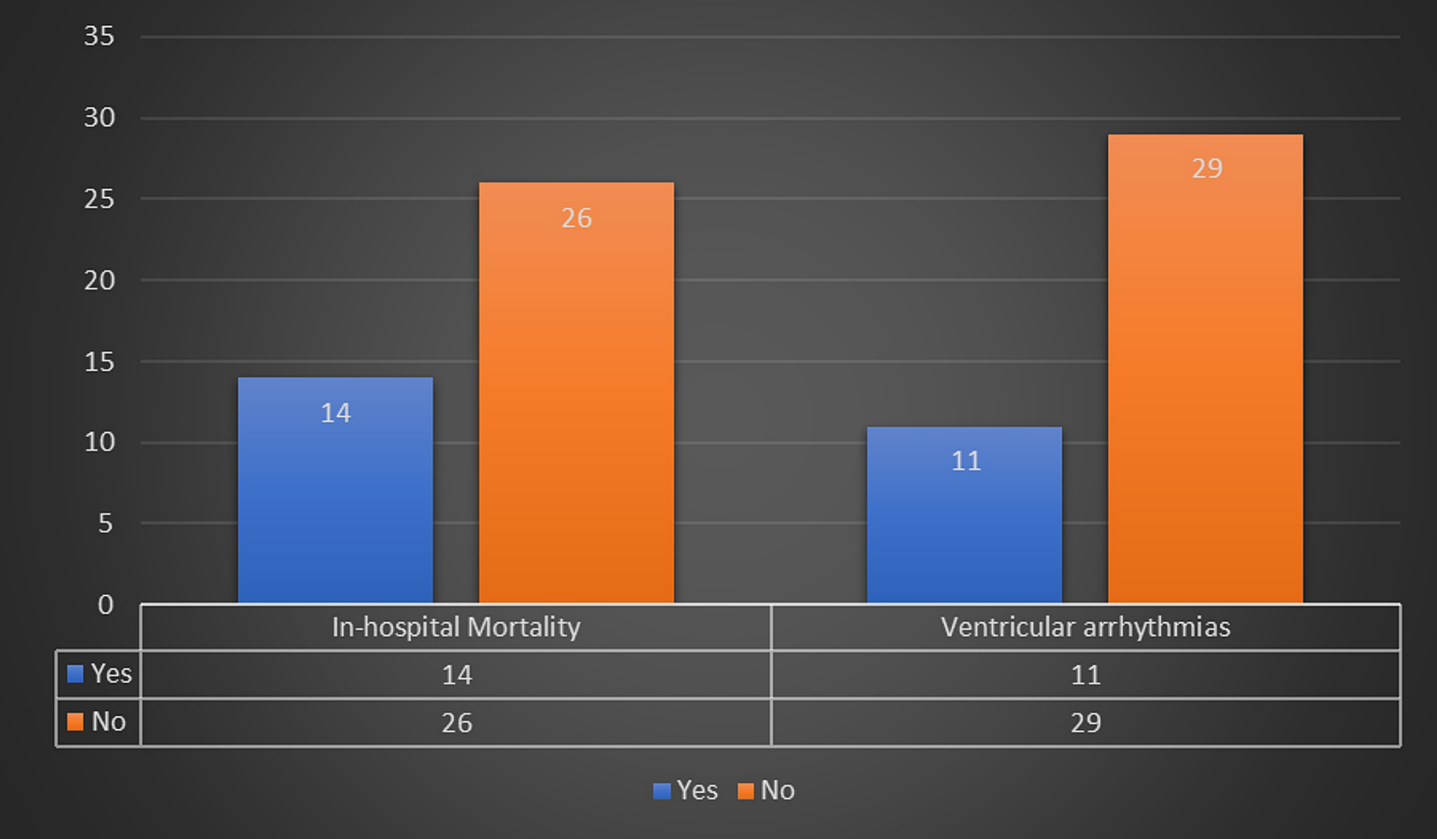
A depiction of frequencies of in-hospital mortality and ventricular arrhythmias.

A total of 11 (27.5%) patients developed VAs and 14 (35%) patients succumbed to the disease complications. These findings are depicted in Figure 1.

The Spearman’s correlation showed a significant positive correlation of prolonged QTc interval with the development of VAs and in-hospital mortality. The values of the correlation coefficient (rho) are tabulated in Table 4.

**Table 4:** Correlation of QTc with in-hospital mortality and ventricular arrhythmias. QTc: corrected QT interval

Parameters	Spearman correlation rho value (with QTc)	P-value
In-hospital mortality	0.314	0.04
Ventricular arrhythmias	0.658	<0.001

## Discussion

VAs are a frequent cause of mortality among patients with STEMI. The factors that predispose STEMI patients to an arrhythmogenic state include partial myocardial revascularization, prolonged chest pain, and genetic predisposition [3]. Additionally, QTc interval prolongation due to an inherent or congenital defect in cardiac channels also plays a fundamental role in contributing to the genetically determined arrhythmogenic state in STEMI [5,7]. This is due to altered electrolyte milieu, disruption of ion channels, and exacerbated sympathetic activity in patients with STEMI [9]. Therefore, careful electrocardiographic and systemic evaluation of such patients remains pivotal to prevent adverse outcomes.

Literature reported that STEMI is more strongly associated with arrhythmias and a plethora of the patients develop VAs within the first hour [4]. Similarly, the results from a case-control study showed that STEMI patients with ventricular fibrillation had prolonged QTc interval than the patients without ventricular fibrillation [14]. Furthermore, the same study reported VAs to be an alarming situation in STEMI with prevalence hovering at 6-10% and requiring prompt intervention [14]. The difference in the prevalence of VAs might be due to the difference in management strategies as most patients in our clinical setting received thrombolytic therapy that significantly elevates the risk of reperfusion arrhythmias [14]. Moreover, Literature reports that patients receiving thrombolytic therapy have an increased likelihood of developing VAs than patients that received reperfusion therapy via PCI [9]. Nonetheless, VAs can still occur during the acute phase of STEMI even with early and effective reperfusion therapy [15]. This illustrates that the QTc interval is an important risk factor of VAs in STEMI patients leading to an increased mortality rate.

It has been elucidated that in-hospital mortality due to VAs in STEMI patients is around 27% [16]. Another study reported a significantly elevated risk of in-hospital mortality among patients with VAs during an acute phase of myocardial infarction [17]. Additionally, a case-control study concluded that QTc interval prolongation increases the risk of mortality from 5% to 20% in STEMI patients [18]. This illustrates that patients with STEMI and QTc interval prolongation are nearly four times more likely to develop adverse outcomes. In comparison, our study showed a higher mortality rate hovering at an alarming 35%. A clinical setting of a developing country, poor utilization of resources, and delayed diagnosis of STEMI might have contributed to the higher mortality rate in our study.

The results of a prior study reported a strong significant correlation between prolonged QTc interval and severity of STEMI (rho = 0.929, p < 0.001) [19]. The increased severity predisposes the patients to develop adverse outcomes including VAs and ultimately higher in-hospital mortality [19]. This necessitates the need for regular monitoring of STEMI patients with QTc interval prolongation. Periodic monitoring with relatively inexpensive and non-invasive ECG and timely cardioversion will help in preventing these adverse outcomes. The therapeutic options available for managing VAs include anti-arrhythmic drugs (amiodarone and lidocaine), over-riding pacing, antiplatelet/anticoagulation therapy radiofrequency ablation, timely coronary intervention, and defibrillator [3].

A limitation of our study was that we utilized registered data which is accustomed to selection bias. Moreover, the time of the VAs was not recorded with respect to the time of reperfusion therapy. Thus, we could not sort through the patients, whether they developed VAs before, during, or after reperfusion therapy. We did not specify the type of VAs our patients developed. Different types of VAs such as ventricular tachycardia and ventricular fibrillation might have different prognostic value concerning STEMI. Nevertheless, the results of our study must be given serious considerations as STEMI is a leading cause of morbidity and mortality, especially in developing countries. There is an utmost need for curation of specific guidelines that help in the management of these high-risk patients so that VAs and in-hospital mortality can be avoided. This will aid in improving the disease management protocols and lower the overall mortality rate due to STEMI. 

## Conclusions

Prolonged QTc interval is positively correlated with VAs and in-hospital mortality among STEMI patients. Risk stratification, early diagnosis, and prompt intervention are extremely important to prevent in-hospital mortality among STEMI patients with QTc interval prolongation. Further studies are required to create a predictive model for better risk stratification of such patients. This necessitates the need for better management strategies and curation of appropriate evidence-based guidelines so that the incidence of VAs can be reduced and adverse in-hospital outcomes can be avoided.
